# Identification of Multiple Bowen's Disease Skin Lesions by Careful Physical Examination in a Patient With Fanconi Anemia

**DOI:** 10.7759/cureus.50016

**Published:** 2023-12-06

**Authors:** Hirofumi Kawamoto, Taiyo Hitaka, Natsuko Saito-Sasaki, Etsuko Okada, Yu Sawada

**Affiliations:** 1 Dermatology, University of Occupational and Environmental Health, Kitakyushu, JPN

**Keywords:** surgical resection, multiple skin tumors, fanconi anemia, dermoscopy, bowen’s disease

## Abstract

Because Fanconi anemia is a hereditary bone marrow failure disease caused by DNA repair dysfunction, malignant skin tumors have been recognized in patients with Fanconi anemia. Herein, we report a 32-year-old male with Fanconi anemia presenting multiple Bowen’s disease skin lesions. He first recognized skin eruption in his scrotum, which was diagnosed with Bowen's disease by dermoscopy examination and histological analysis. Due to the elevated risk of skin cancers in Fanconi anemia, we conducted additional meticulous examinations using dermoscopy on the entire body's skin, revealing another skin tumor on his back. A skin biopsy confirmed the diagnosis of another site of Bowen's disease. Therefore, additional thorough examinations using dermoscopy might aid in identifying multiple skin tumors in high-risk cases of skin malignancies, such as Fanconi anemia.

## Introduction

Fanconi anemia is a hereditary bone marrow failure disease and is characterized by chromosomal instability caused by the dysfunction of DNA repair [1,2]. Pancytopenia, skin pigmentation, congenital anomalies, and gonadal dysfunction are representative clinical characteristics observed in this clinic. Although generalized pigmentation or café au lait patches (40%) and depigmentation (35%) are typical skin symptoms [3], malignant skin cancers have been identified in patients with Fanconi anemia [4]. These findings indicate that patients with Fanconi anemia are at a higher risk of developing skin cancer. However, the actual process of identifying these multiple skin cancers has not been described in a limited number of previous case studies [5]. Herein, we report a case of multiple Bowen’s disease skin lesions in a patient with Fanconi anemia, which was identified by careful physical examinations and histological analyses.

## Case presentation

A 32-year-old male was diagnosed with Fanconi anemia based on the presence of pancytopenia, polydactyly of the right thumb, deformation of the left auricle, strabismus, skin pigmentation, short stature, and a chromosomal test indicating fragility due to the addition of mitomycin C. At the age of 17, he underwent bone marrow transplantation and received treatment with tacrolimus. Additionally, at the ages of 25 and 31, he underwent surgical resection for hypopharyngeal squamous cell carcinoma and oral squamous cell carcinoma, respectively. He had no experience of the recurrence or distant metastasis of both squamous cell carcinomas. He also received treatment for hyperlipidemia with rosuvastatin and allergic rhinitis with montelukast and levocetirizine. He had no history of smoking.

**Figure 1:**
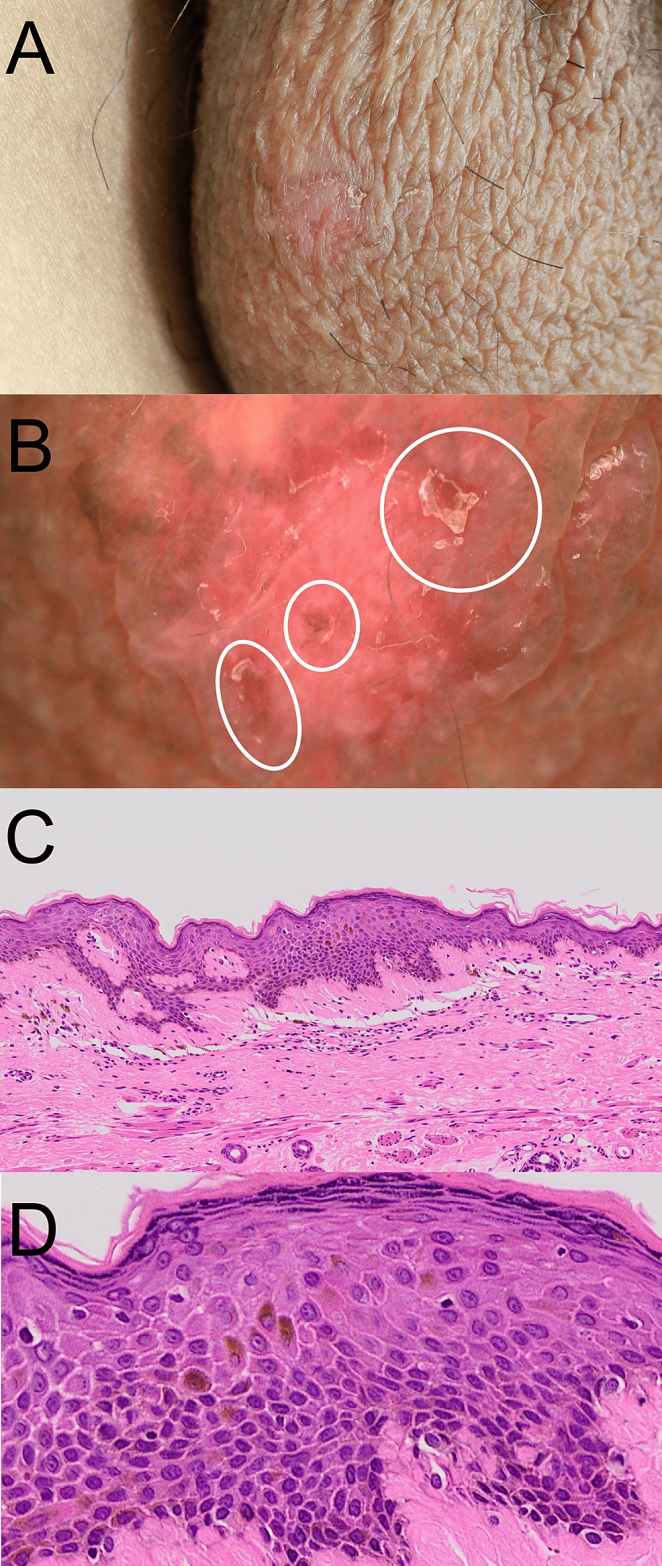
Clinical and histological findings of the scrotum skin tumor. (A) Clinical appearance; (B) dermoscopy examination; white circles indicate scaly surfaces; (C, D) histological investigation: (C) a view magnified at x40 and (D) a view magnified at x400.

He noticed a brownish scaly plaque on his scrotum and was referred to our department for evaluation. A physical examination showed a scaly erythematous skin eruption on his scrotum (Figure 1A). A dermoscopy examination revealed an erythematous scaly surface (Figure 1B). A skin biopsy taken from this skin eruption showed atypical epidermal keratinocytes with a high nucleus-to-cytoplasmic (N/C) ratio throughout the epidermal layer, confirming the diagnosis of Bowen’s disease (Figures 1C-1D).

**Figure 2:**
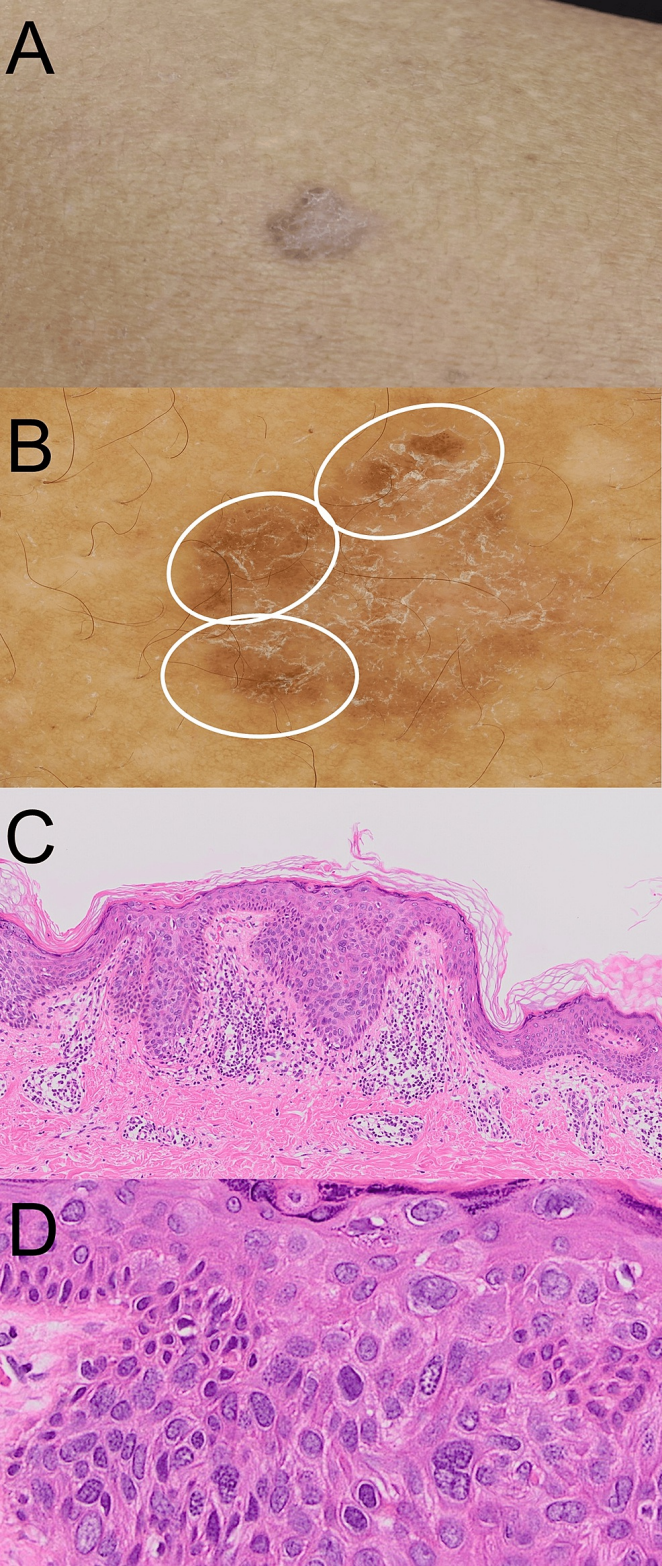
Clinical and histological findings of the back skin tumor. (A) Clinical appearance; (B) dermoscopy examination; white circles indicating brown structureless areas; (C, D) histological investigation: (C) a view magnified at x40 and (D) a view magnified at x400.

Due to his previous histories of squamous cell carcinoma in other organs, specifically hypopharyngeal and oral squamous cell carcinoma, we suspected the presence of additional skin tumor sites. A scaly, brown, elevated lesion and erythema were seen in his left upper back (Figure 2A). A dermoscopy examination showed a scaly surface and brown structureless areas (Figure 2B). A skin biopsy taken from the eruption showed a mild elongation of epidermal protrusions, with inflammatory cells infiltrating the dermis (Figure 2C). There were clumping cells with dysplasia, and these cells showed a high N/C ratio, leading to the diagnosis of Bowen’s disease (Figure 2D).

No recurrence of the tumor was observed after the surgical resection.

## Discussion

Various malignant tumors have been identified in patients with Fanconi anemia. It is known that the overall risk of malignant tumor occurrence is around 50 times higher than that of healthy subjects [3]. Skin cancer is typically observed at a young age in patients with Fanconi anemia [4]. The average onset age for squamous cell carcinoma and basal cell carcinoma in patients with Fanconi anemia is earlier compared to healthy subjects, at 27 and 25 years, respectively. Therefore, careful observation is essential even at a young age, and the potential for multiple skin lesions, as seen in this case, should be considered during monitoring.

Fortunately, we conducted a thorough examination of the entire body's skin and identified multiple Bowen's disease, a precancerous squamous cell carcinoma lesion. In cases with a high risk of skin cancers, a comprehensive physical examination of the entire body may be beneficial for identifying additional skin cancer sites.

In addition, in this case, the hypopharyngeal squamous cell carcinoma exhibited positivity for HPV in the tumor. Patients with Fanconi anemia exhibit a high frequency of HPV infection [6]. The hypopharyngeal cancer was positive for anti-HPV antibodies (clone K1H8), suggesting a possible influence of HPV infection in this patient. However, both Bowen’s disease lesions yielded a negative result for HPV in an immunohistochemical examination, suggesting a non-viral-mediated occurrence of the tumors in the skin.

The incidence of solid cancer increases after bone marrow transplantation [7]. Consistently, patients who underwent bone marrow transplantation were nearly 40 times more likely to develop skin cancer compared to the general healthy population [7]. The multiple occurrences of Bowen's disease in this case are thought to possibly be due to the disorder of the DNA repair mechanism in Fanconi anemia. In addition, the history of bone marrow transplantation and associated immunosuppressive drug use may also influence the occurrence of Bowen's disease.

## Conclusions

Based on our experience, a whole-body skin examination using dermoscopy proved useful in identifying other skin tumor sites. Given the higher risk of malignancies in cases of skin tumors, a thorough physical examination of the entire body's skin becomes essential. In addition, Fanconi anemia remains an unfamiliar disease entity for dermatologists. Therefore, clinicians should keep in mind that Fanconi anemia is one of the representative high-risk diseases associated with the occurrence of malignant skin cancers.
